# Visual fixations rather than saccades dominate the developmental eye movement test

**DOI:** 10.1038/s41598-020-80870-5

**Published:** 2021-01-13

**Authors:** Nouk Tanke, Annemiek D. Barsingerhorn, F. Nienke Boonstra, Jeroen Goossens

**Affiliations:** 1 Department of Cognitive Neuroscience, Donders Institute for Brain, Cognition and Behaviour, Radboud University Medical Centre, Nijmegen, The Netherlands; 2 Department of Biophysics, Donders Institute for Brain, Cognition and Behaviour, Radboud University, Nijmegen, The Netherlands; 3 Royal Dutch Visio, National Foundation for the Visually Impaired and Blind, Nijmegen, The Netherlands

**Keywords:** Oculomotor system, Visual system

## Abstract

When children have visual and/or oculomotor deficits, early diagnosis is critical for rehabilitation. The developmental eye movement (DEM) test is a visual-verbal number naming test that aims to measure oculomotor dysfunction in children by comparing scores on a horizontal and vertical subtest. However, empirical comparison of oculomotor behavior during the two subtests is missing. Here, we measured eye movements of healthy children while they performed a digital version of the DEM. In addition, we measured visual processing speed using the Speed Acuity test. We found that parameters of saccade behavior, such as the number, amplitude, and direction of saccades, correlated with performance on the horizontal, but not the vertical subtest. However, the time spent on making saccades was very short compared to the time spent on number fixations and the total time needed for either subtest. Fixation durations correlated positively with performance on both subtests and co-varied tightly with visual processing speed. Accordingly, horizontal and vertical DEM scores showed a strong positive correlation with visual processing speed. We therefore conclude that the DEM is not suitable to measure saccade behavior, but can be a useful indicator of visual-verbal naming skills, visual processing speed, and other cognitive factors of clinical relevance.

## Introduction

Visual deficits that are not directly related to an abnormality in the peripheral visual system, but rather to an injury in brain areas that play a role in the perception or interpretation of visual information, are often difficult to recognize (for a review, see^1^). Especially children born with visual deficits might not realize that their perception of the world is distorted. However, clinical tools to diagnose deficiencies in visual information processing and oculomotor functioning at an early age are limited^2^. Apart from visual and or oculomotor dysfunction, the child can show attentional or cognitive deficits, higher cortical visual processing, or language retrieval issues too^3,4^.

**Figure 1:**
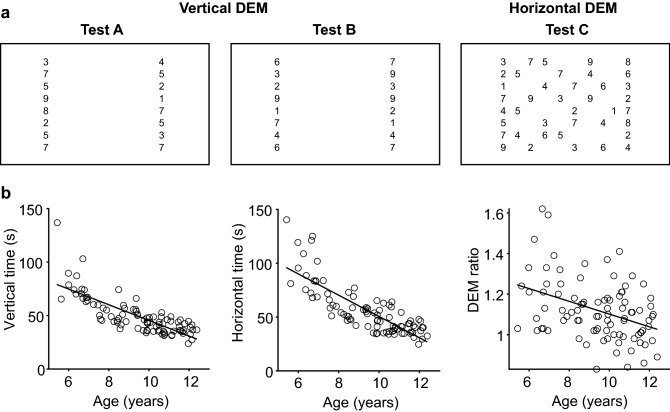
Developmental Eye Movement test (DEM) improves with age. (**A**) Partial representation of the digital DEM (not drawn to scale for clarity; see methods for details). Three arrays of numbers are subsequently shown on a computer screen, separated by short breaks. Part A and B each consist of two columns of 20 numbers that have to be read out loud from top to bottom (vertical time: combined time for test A and B). Part C consists of 16 rows of five numbers that have to be read out loud from left to right, starting at the top left corner (horizontal time). (**B**) Completion time adjusted for errors decreases with age, for the vertical DEM (left) and the horizontal DEM (middle). The ratio of horizontal to vertical time (DEM ratio, right) also decreases with age. All *p* values < 0.001. Produced in MATLAB 2018b (www.mathworks.com) and Adobe Illustrator CC 2017 (www.adobe.com).

The Developmental Eye Movement test (DEM) is a visual-verbal reading task originally developed to assess oculomotor function in children without the use of eye tracking techniques^5^. In this test, subjects first read out loud a standardized list of vertically arranged numbers and then name numbers in a two-dimensional array row by row (Fig. 1a). The vertical subtest primarily calls for small equally spaced vertical saccades, whereas the horizontal subtest requires horizontal saccades of varying magnitude. The assumption is that, by eliminating the requirement for horizontal saccades of varying amplitude, the physiological constraints on the oculomotor system are reduced in such a way that the vertical subtest is predominantly determined by automatic visual-verbal naming skills (automaticity). According to this assumption, performance on the vertical subtest can be used to factor out the effects of automaticity in the horizontal subtest. An increase in both horizontal and vertical reading time compared to age-matched controls would thus indicate a difficulty in general number naming skills, which in healthy children dominates between 64 and 90% of the time spent in horizontal DEM^6^. An abnormally increased time on the horizontal test combined with a normal performance on the vertical test (a high DEM ratio) would be characteristic of oculomotor dysfunction. However, Garzia et al.^5^ did not measure eye movements or verified otherwise whether the horizontal to vertical ratio truly reflect oculomotor skills.

Therefore, the DEM’s use for the assessment of oculomotor function has been questioned. Test–retest reliability of the DEM ratio is only moderate to low^7–9^. In addition, DEM performance does not correlate significantly with parameters of horizontal saccades measured in saccade tasks that require no numerical processing or verbalization^10^. Children with amblyopia (where one of the symptoms is a poorer efficiency in oculomotor control) are also not affected in their DEM performance^11^. These results suggest poor specificity and sensitivity of the DEM ratio in measuring oculomotor skills and detecting oculomotor deficiencies.

Nevertheless, horizontal and vertical DEM times do show good repeatability in test–retest studies^7–9,12^. Both subtests are also a good indication of the level of academic performance^5,13,14^, reading rate^6,15–17^, and speed of visual processing^10^. Additionally, children with dyslexia perform worse than typically developing readers^18^. For these reasons, the horizontal and vertical DEM can still be useful in clinical settings when a quick assessment of visual functioning is required^10,19^.

To investigate whether the DEM could still be useful in the assessment of oculomotor function, it is important to measure eye movements during the DEM subtests. Webber et al.^19^ used goggles containing infrared sensors to study eye movement patterns during a reading task, and compared those results to DEM scores. They found that the horizontal DEM, but not the vertical DEM or the DEM ratio, correlated significantly to the number and average duration of fixations in the reading task. To the best of our knowledge, Ayton et al.^10^ and Moiroud et al.^20^ were the only groups to measure eye movements during the horizontal DEM. Both studies found a significant correlation between fixation durations and the time needed to complete the test. However, by analyzing only fixation durations during the horizontal DEM, it remains unclear whether saccade behavior is related to overall DEM performance.

To fill this gap, we recorded eye movements of healthy children with normal vision while they performed a digital version of the horizontal and vertical DEM. As far as we know, the present study is the first to measure horizontal and vertical eye movements in both subtests. To relate DEM performance to visual processing speed, subjects were tested in a speed acuity task as well (SA;^21^). This SA task measures visual acuity and visual discrimination speed simultaneously, and can detect subtle anomalies in visual processing^22^.

## Results

The DEM (Fig. 1a, Supplementary Fig. S1) was administered on a computer screen at a viewing distance of 65 cm to allow for head-free eye-tracking with a remote eye-tracking system^23^. Size and spacing of the numbers were adjusted to the viewing distance to match the original version of the DEM taken at 33 cm. Like the original DEM^5^, this digital version also consisted of three parts. Test A and B had to be read aloud from top to bottom and test C had to be read aloud from left to right. Vertical time was the time needed for test A and B together, horizontal time was the time needed to complete test C. To evaluate performance, horizontal time was adjusted for errors in number identification such as number omissions (see methods for details). Most participating children (84/91) made two errors or less (Supplementary Fig. S2A). Younger children made more errors than older children did. Horizontal times for children with more than two errors were comparable to the horizontal times of age-matched children who made two errors or less (Supplementary Fig. S2B). We therefore decided not to exclude any children based on the number of errors made.

### DEM scores are age-dependent

To determine test-validity of the digital DEM, we studied the relationship between age and DEM time in all 91 participants. In line with previous studies, younger children (6–7 years old) needed more time to complete either DEM compared to older children (11–12 years old). We found a significantly negative correlation between age and vertical time (r = − 0.79), age and horizontal time (r = − 0.81) and age and DEM ratio (r = − 0.39 all *p *values < 0.001; Fig. 1b). The latter suggests an increasing dominance of the naming process in horizontal DEM with age. The mean and standard deviations per age group Table 1) fell within the mean ± SD given in previous reports^5,6,8^. This indicates that the digital DEM can be used in addition to or as a replacement of the original DEM. Only the numbers of errors were lower in our study population. We think this is because we allowed children to briefly practice each subtest (with different numbers), instead of just showing an example row and explaining the task in words (Methods). We did this to ensure a correct understanding of the tasks.

**Table 1:** Average DEM scores per age group. Scores averaged across all participants. Horizontal DEM times are adjusted for the number of errors. Naming is vertical DEM time (in sec) as a percentage of horizontal DEM time (in sec).

Age	Vertical DEM mean (SD)	Horizontal DEM mean (SD)	DEM ratio mean (SD)	Errors mean (SD)	Naming (%) mean (SD)
6 (n = 12)	73.42 (11.39)	89.39 (19.25)	1.22 (0.21)	2.08 (2.28)	84% (13.48)
7 (n = 7)	53.51 (6.12)	64.20 (10.61)	1.20 (0.11)	1.14 (1.22)	84% (7.83)
8 (n = 12)	49.56 (9.96)	55.66 (10.78)	1.13 (0.11)	0.67 (0.98)	89% (8.40)
9 (n = 16)	46.01 (9.05)	49.37 (9.61)	1.08 (0.12)	0.44 (0.72)	94% (10.27)
10 (n = 18)	40.42 (6.67)	44.90 (10.95)	1.10 (0.14)	0.39 (0.70)	92% (11.99)
11 (n = 16)	38.03 (6.68)	39.56 (7.17)	1.04 (0.11)	0.38 (1.26)	97% (9.94)
12 (n = 6)	35.57 (4.99)	37.61 (5.98)	1.06 (0.11)	0.50 (0.55)	95% (10.73)

### Eye movements during horizontal and vertical DEM

Next, we analyzed the saccade behavior during the horizontal and vertical DEM to determine how oculomotor skills influence the time needed to accomplish these subtests. Eye-tracking data were not available for all children, as it is difficult to obtain complete data sets in this particular population. For 20/91 children, no eye movements were recorded owing to technical problems, children that could not sit still enough, or problems with detecting the eyes (e.g., due to problematic glasses). For 35/91 children, signals were lost too often for analysis (in most cases because the child moved outside the view of the eye tracker). We successfully measured eye movements for the entire duration of the horizontal DEM in 36 children (10.2 ± 1.4 years). Horizontal and vertical DEM times in this subpopulation were similar to those of the entire population (Supplementary Table S1). For the vertical DEM, we obtained eye movement data from 28/36 children. Complete records during both test A and test B were obtained in 16/28 children. For 12/28 children, complete records were successfully obtained for either test A or test B. In those cases, we duplicated the DEM A or DEM B time as well as the relevant oculomotor parameters (fixation time, saccade time, and number of eye movements) to allow for comparison with the other datasets. This correction is justified by the observation that two times the performance time of DEM A or DEM B did not differ from the sum of the two in this dataset [t(11) = 1.94, *p* = 0.079, paired *t* test]. Most tracking problems occurred in young children. Consequently, there is a slight bias in the data towards older children. The youngest child whose data could be included was 7.2 years old.

**Figure 2:**
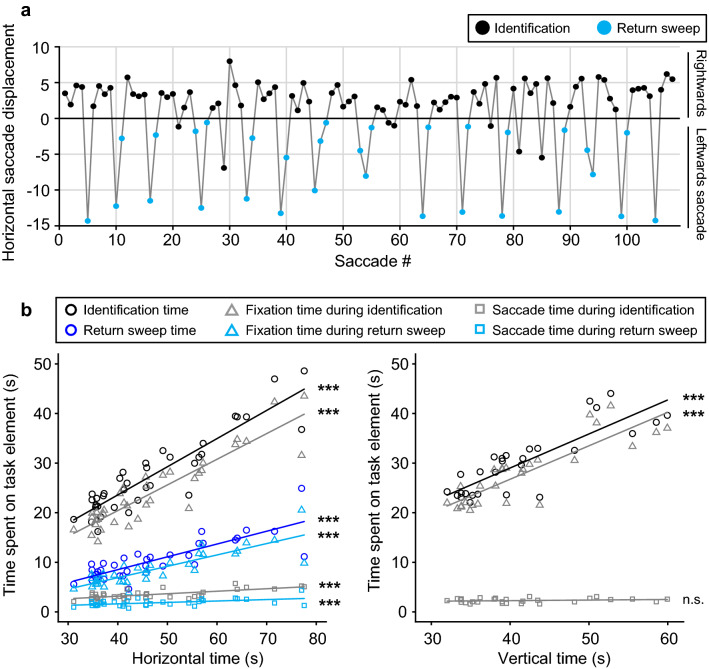
Oculomotor behavior during DEM testing. (**A**) Horizontal eye displacement for all subsequent saccades of a representative child during execution of the DEM C. Each dot represents a saccade. Positive displacement: saccade to the right, negative displacement: saccade to the left. Black dots: saccades during number identification. Blue dots: saccades made during return sweeps back to the start of a new line. (**B**) Time spent on different parts of the DEM task plotted against the total time needed to complete the task without correction for errors (left, horizontal time; right, vertical time). Circles: total time spent on number identification (black) and sweeping back to the beginning of the next line (blue). Triangles: total time spent on fixation during number identification (gray) and returning to the next line (light blue). Squares: total time needed for saccades during number identification (gray) and return sweeps (light blue) ***p < 0.001, n.s. = not significant. Produced in MATLAB 2018b (www.mathworks.com).

Using the gaze position signals, we separated the horizontal DEM into different epochs: “number identification epochs” and “return sweep epochs”. Number identification epochs were taken from the moment the point of gaze arrived at the beginning of a line until the point of gaze arrived at the end of a line and the eye started moving towards the beginning of another line. Return sweep epochs were taken from the moment the point of gaze started moving from a line ending towards the beginning of another line until the point of gaze arrived at the beginning of another line, with or without intermittent fixations. Figure 2a shows the horizontal displacement of the right eye of a representative child during the horizontal DEM (see Supplementary Fig. S3 for raw eye-tracking data of the same child). Note that, instead of one swift saccade to the beginning of the next line, children often made multiple smaller saccades to reach the beginning of the next line.

Figure 2b shows how much time subjects spent on different task elements in relation to the total time they needed to complete the two subtests (uncorrected for naming errors). We first looked at how much time subjects needed for number identification (i.e., all number identification epochs combined) compared to making the return sweeps (i.e., all return sweep epochs combined) during the horizontal DEM. As expected, subjects spent more time on number identification than on return sweeps [t(35) = 17.33, *p* < 0.001, paired-sample *t* test]. This is also clear from Fig. 2b, left, where the regression line for total return sweep time (blue circles; r = 0.82, *p* < 0.001) falls far below the line for total number identification time (black circles; r = 0.91, *p* < 0.001). Next, we parsed the number identification epochs and return sweep epochs into time spent on number fixation (triangles; fixation time) and time spent making saccades (squares; saccade time) by summing the durations of fixation periods and saccades occurring within those time windows, respectively. Although saccade time did correlate significantly with overall performance, for both the identification (r = 0.70, *p* < 0.001) and return sweep epochs (r = 0.54, *p* < 0.001), the largest contribution to the horizontal DEM was the time spent on fixation during the number identification epochs (identification, r = 0.91, *p* < 0.001; return sweep, r = 0.85, *p* < 0.001). A similar pattern was found for the vertical DEM (Fig. 2b, right). For this subtest, we observed a strong positive correlation between total fixation time during the number identification epochs and overall performance too (r = 0.85, *p* < 0.001) and noticed that only a small fraction of the time involved making saccades. However, saccade time during number identification was not significantly correlated with the time needed to complete the vertical DEM (r = 0.21, *p* = 0.28).

**Figure 3:**
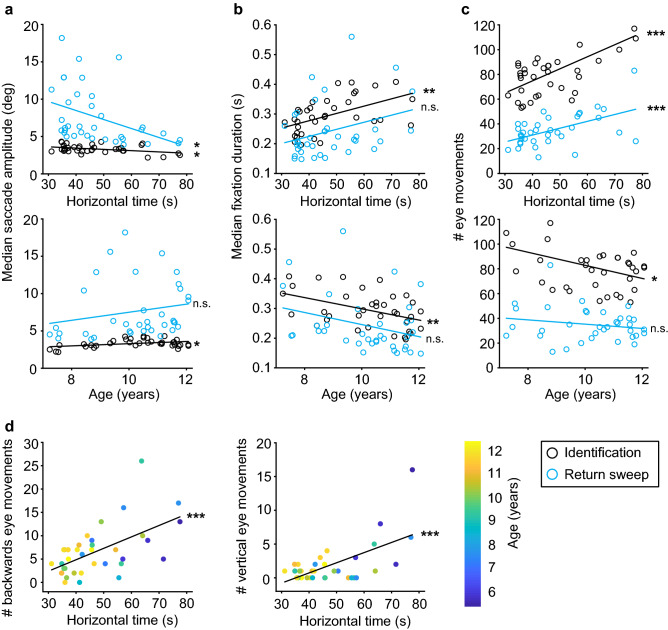
Saccade amplitude and eye movement counts during horizontal DEM testing. (**A**) Median saccade amplitude during number identification (black) and return sweeps (blue) plotted against horizontal DEM (top) and age (bottom). (**B**) Same as A, but for median fixation duration. (**C**) Same as A, but for number of eye movements. (**D**) Number of backwards eye movements (left) and number of vertical eye movements (right) during number identification of the horizontal DEM. Color-coded for age (colorbar right). ***p < 0.001, **p < 0.01, *p < 0.05, n.s. = not significant**.** Produced in MATLAB 2018b (www.mathworks.com).

To explore further what made some children more efficient than others, we plotted the median saccade amplitude, the median fixation duration and the number of eye movements made as a function of horizontal DEM time and age. Because developing readers make more corrective saccades during return sweeps than adults^24^, one would expect, for instance, that children with better DEM performance make fewer but larger saccades during the return sweep epochs. Saccade amplitude increased significantly with decreasing horizontal DEM time, both during return sweeps epochs (Fig. 3a top; r = − 0.41, *p* = 0.014) and during number identification epochs (r = − 0.40, *p* = 0.014). The median saccade amplitude during number identification epochs also increased significantly with age (Fig. 3a bottom; r = 0.39, *p* = 0.018), but the median amplitude of return sweep saccades was not age-dependent (r = 0.21, *p* = 0.23). Based on previous research^10,20^, we expected a decrease in fixation durations with improved performance. The median fixation duration indeed decreased with improving DEM times but only during the number identification epochs (Fig. 3b top; r = 0.52, *p* = 0.0013), not during the return sweep epochs (r = 0.32, *p* = 0.059). These results were age-related, because the median fixation duration decreased with age albeit not statistically significant for the return sweep epochs (Fig. 3b, bottom; number identification: r = − 0.45, *p* = 0.0063; return sweep: r = − 0.31, *p* = 0.070). These findings show that the amplitudes of return sweep saccades increased with improved DEM performance, while the durations of intermittent fixations were unaltered. For number identification periods, on the other hand, the saccade amplitudes increased and the fixation durations decreased.

As one might expect, the changes in saccade amplitude were paralleled by changes in number of saccades. A lower number of saccades was associated with an improved horizontal DEM time, both during return sweep epochs (Fig. 3c top; r = 0.53, *p* < 0.001) and also during number identification epochs (r = 0.66, *p* < 0.001). The number of eye movements also decreased with age for the number identification epochs (Fig. 3c bottom; r = − 0.40, *p* = 0.015), but not for the return sweep epochs (r = − 0.19, *p* = 0.27). In line with these findings, we expected more backwards saccades and more vertical saccades during number identification epochs in children with longer horizontal DEM times. Indeed, we found significantly positive correlations between horizontal DEM time and the number of backwards eye movements (Fig. 3d top; r = 0.58, *p* < 0.001) and the number of vertical eye movements (Fig. 3d bottom; r = 0.63, *p* < 0.001).

The vertical DEM limits the use of return sweeps and horizontal saccades. We wondered how saccade behavior differed between the horizontal and vertical DEM as the test relies on the comparison between these subtests to evaluate oculomotor performance. We therefore determined the relationship between vertical DEM and the oculomotor parameters as analyzed above for horizontal DEM. In this case, we focused on the number identification periods because the vertical DEM only calls for two return sweeps, one per subtest. We found no significant association between vertical DEM time and saccade amplitude, number of eye movements, or number of backwards saccades (|r|< 0.35, *p* > 0.07; for details, see Supplementary Fig. S4). However, there was a strong positive correlation between vertical DEM time and median fixation duration (Supplementary Fig. S4B top; r = 0.75, *p* < 0.001), as well as a decrease in fixation durations with age (Supplementary Fig. S4B, bottom; r = − 0.52, *p* = 0.0049).

**Figure 4:**
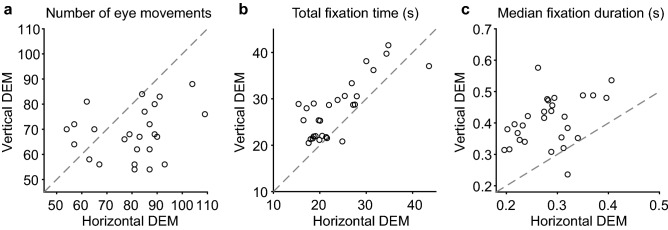
Number of eye movements, total fixation times and fixation durations in horizontal versus vertical DEM. Number identification epochs only. Grey dashed lines represent X = Y so outcomes should fall on this line if there is no difference between the horizontal (x-axis) and vertical (y-axis) DEM. (**A**) Total number of eye movements. (**B**) Total fixation time (i.e., sum of fixation durations). (**C**) Median fixation duration. Produced in MATLAB 2018b (www.mathworks.com).

Figure 4 characterizes the differences in oculomotor behavior between the vertical and horizontal DEM. Note, that more saccades were made during the number identification epochs in horizontal DEM than in vertical DEM [Fig. 4a, t(27) = 3.32, *p* = 0.0026, paired *t* test]. Additionally, both the total fixation time and the median fixation durations measured during the number identification epochs were significantly longer in the vertical DEM than in the horizontal DEM [Fig. 4b, total fixation duration, t(27) = 5.20, *p* < 0.001; Fig. 4C, median fixation duration, t(27) = 8.53, *p* < 0.001, paired *t* test]. As expected because of the smaller spacing between numbers in the vertical versus horizontal DEM, the median saccade amplitudes were significantly smaller in the vertical DEM compared to the horizontal DEM [data not shown; t(27) = 21.11, *p* < 0.001]. We also detected more orthogonal eye movements during the horizontal DEM than during the vertical DEM [data not shown, t(27) = 3.31, *p* = 0.0027]. However, the number of backwards saccades did not differ significantly between the horizontal and vertical DEM [data not shown, t(27) = 1.85, *p* = 0.076, paired *t* test]. Overall, Fig. 4 indicates a difference in the use of eye movements during number identification in the two subtests.

### DEM and the Speed Acuity task

**Figure 5:**
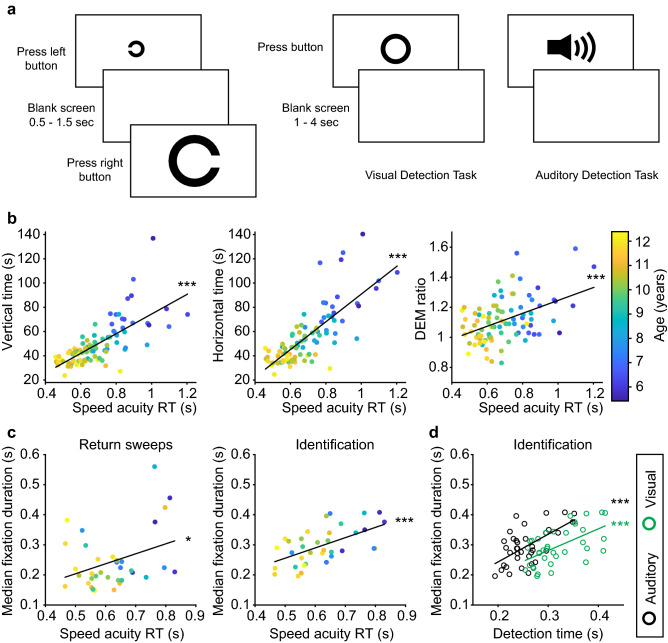
DEM performance versus visual processing speed. (**A**) Visual representation of the Speed Acuity task (SA; left) in which subjects have to quickly and accurately discriminate the orientation of a Landolt C, and the detection tasks (right) in which subjects have to respond quickly to the occurrence of a suprathreshold visual or auditory stimulus. (**B**) Reaction time during the SA plotted against vertical DEM (left), horizontal DEM (middle) and DEM ratio (right), color-coded for age (colorbar right). DEM scores are adjusted for errors. (**C**) Similar to A, but plotted against median fixation duration during return sweeps (left) and during identification (right). (**D**) Median fixation duration during identification plotted against reaction times in the visual detection task (green) and an auditory detection task (black). ***p < 0.001, *p < 0.05. Produced in MATLAB 2018b (www.mathworks.com) and Adobe Illustrator CC 2017 (www.adobe.com).

To assess the contribution of visual information processing time to the DEM, we compared DEM times to reaction times measured during a Speed Acuity test (SA) in 90/91 children who performed both the DEM and the SA (see Supplementary Table S2 for details). The SA measures visual processing speed for different symbol sizes^21^. In this task, children had to discriminate the orientation of a Landolt “C” as left or right by pressing the corresponding mouse button (Fig. 5a, left). Besides this discrimination task, each child also performed a visual and an auditory detection task (Fig. 5a, right). We compared the DEM performance to mean reaction time during the SA for matched symbol sizes, and found significantly positive correlations between the SA reaction times and performance in the DEM (Fig. 5b; vertical DEM r = 0.71, *p* < 0.001; horizontal DEM r = 0.77, *p* < 0.001; DEM ratio r = 0.45, *p* < 0.001). Note that, similar to the DEM measures, SA scores correlated significantly with age (r = − 0.79, *p* < 0.001). This implies that age could be the confounding factor in the relationship between DEM and SA. However, when age was taken into account as a covariate, the correlations between SA and all aspects of the DEM remained statistically significant (partial correlation coefficient: horizontal DEM rho = 0.35, *p* < 0.001; vertical DEM rho = 0.23, *p* = 0.027; DEM ratio rho = 0.23, *p* = 0.029).

Figure 2 shows that the total fixation time during number identification contributes strongly to horizontal DEM performance. Together with the results in Fig. 5b, this raised the question whether fixation durations correlate with SA performance. Indeed, we found a strong positive correlation between SA and median fixation duration during number identification epochs (Fig. 5c left; right; r = 0.58, *p* < 0.001) and a weaker correlation during return sweeps (r = 0.36, *p* = 0.036). Additionally, if horizontal DEM time is a good indication of visual processing speed, we should see a stronger relationship to performance in a visual detection task than to performance in a similar detection task with an auditory stimulus. However, fixation durations during number identification correlated equally well to performance in the auditory detection task (Fig. 5d; visual detection: r = 0.58, *p* < 0.001; auditory detection: r = 0.60, *p* < 0.001; difference between correlation coefficients, *p* = 0.90). All p-values provided in Fig. 5b–d remained statistically significant after correction for multiple comparisons.

## Discussion

We combined the horizontal and vertical DEM with eye tracking and studied the relation with performance on a visual SA test. Both vertical and horizontal DEM times strongly related to the visual processing speed measured in the SA test as well as the eye fixation durations measured in the DEM. These findings agree with the results of Ayton et al.^10^ and Moiroud et al.^20^ for the horizontal DEM, and extend the results to the vertical DEM. Additionally, we found that faster performance on the horizontal DEM coincided with fewer, but typically larger saccades. Children who needed more time for the horizontal DEM also made more backwards and vertical saccades in the number identification epochs. By contrast, we did not find a significant relationship between the time children needed for the vertical DEM and the number of vertical or horizontal saccades that they made in this subtest. The median amplitude of saccades made during the number identification epochs of the vertical DEM was also unrelated to the time children needed for that test. However, the time that subjects spent on making saccades was very short in comparison to the time they needed to complete either subtest. Instead, most of the variation in either subtest was linked to variation in the number and duration of fixations, which strongly correlated with the participants’ visual processing speed measured in the SA test.

We want to emphasize that visual processing speed is probably one of many factors that influence fixation durations in the DEM. Fixation durations include components relevant to saccade planning^25^, and decreasing horizontal saccade latencies in child development^26^ could explain variations in fixation duration. Fixation durations are also influenced by attentional, verbal and cognitive variables^27^. The developing visual-verbal naming process, for example, could explain decreased fixation times with age^6^. Note, however, that the correlations between visual processing speed and fixation durations remained significant with age as a covariate. A limitation of our study is that we did not asses the naming process in the absence of eye movements using, for example, a rapid serial visual presentation task^28^. However, performance on such tasks is probably influenced by visual processing speed too, making it difficult to dissect the relative contributions.

The King-Devick (K-D) test is another visual-verbal test used for clinical assessment of saccade behaviour and attention^29,30^. It is similar to the horizontal DEM in design, consisting of eight lines instead of sixteen. Performance on the DEM and K-D is indeed strongly correlated^31^ and performance on the K-D test is also positively correlated to fixation duration^32^. Garzia et al. argued, however, that in order to gain knowledge about oculomotor skills, the automaticity of the visual-verbal naming component has to be eliminated^5^. For this reason, they introduced the vertical DEM, assuming that visual-verbal naming automaticity is the dominant factor determining performance on the vertical DEM. The correlation in the time that subjects spent on number identification between the horizontal and vertical DEM as well as the correlations with visual processing speed indeed support the idea that rapid automatic naming skills and visual processing speed play an important role in both tasks^10^.

Interestingly, however, if one discards the time needed for the return sweeps, subjects needed more time naming the numbers in the vertical subtest than in the horizontal subtest. This is inconsistent with the assumption that the vertical DEM accurately reflects the time spent on naming in the horizontal DEM (Table 1, and^6^). Fixation durations were also significantly longer in the vertical DEM. This latter finding is opposite to the assumptions of Garzia et al.^5^, who thought that in the horizontal subtest “saccade latency is greater because of target unpredictability”. How can this be understood? First, we note that all numbers are continuously visible, so the spatial locations of targets are predictable for the duration of the test. Second, in the vertical subtest, the spacing between successive numbers is smaller, which calls, at least in principle, for more precise saccades. The longer fixation durations in the vertical DEM could thus be a consequence of a speed accuracy tradeoff seen in nearly all goal-directed behaviors. In any case, it is known that saccade latencies increase rapidly for targets appearing within 2° of the current fixation^33,34^, and horizontal saccade latencies are significantly shorter than vertical saccade latencies^35^. Lastly, crowding could also play a more prominent role in the vertical DEM compared with the horizontal DEM^36^, thereby impeding the visual verbal naming process and increasing fixation times^6^.

Our finding that the number, direction and amplitude of saccades correlated stronger with the horizontal DEM than the vertical DEM, supports the idea that the horizontal subtest puts a stronger emphasis on saccade behaviour compared with the vertical subtest. Yet, it is incorrect to think that, because of these findings, the comparison between horizontal and vertical DEM times would allow for conclusions regarding oculomotor (dys)function. First, as the purpose of saccades is to rapidly redirect gaze, we found that the contribution of saccade time (the sum of saccade durations) to the total time spent on number identification is small, and almost negligible compared to the overall DEM time. It is not surprising, therefore, that saccade parameters measured under different task conditions did not correlate significantly with horizontal DEM performance^10,19,20^. Second, visual search can take place with or without eye movements^37–39^, as humans are capable of shifting attention without redirecting their eyes. The observed differences in saccade behavior could therefore reflect different visual search strategies imposed by the spatial layout of the numbers, rather than physiological differences between horizontal and vertical saccade programming. Indeed, in the horizontal DEM, but not the vertical DEM, neighboring distractors in the direction orthogonal to the reading direction compete for selection with the target number. This explains the larger number of saccades made in the orthogonal direction during horizontal DEM compared to vertical DEM, whereas the number of backwards saccades was comparable. Finally, the comparison between horizontal and vertical DEM to factor out automaticity implicitly assumes that vertical saccades are generally unaffected in subjects with oculomotor problems. The neurophysiological organization of the saccadic system does not justify this assumption^40^. Specific lesions in the brain stem and cerebellum can selectively affect horizontal saccades as the circuits controlling the horizontal and vertical components of saccades are largely separated at these levels, but this is not the case at the cortical and subcortical planning stages where saccades are represented in topographically organized maps^41,42^.

An important factor for the difference between vertical and horizontal DEM times, and hence to deviations of the DEM ratio from 1, is the time needed for the 15 return sweeps in horizontal DEM. In line with previous research^24^, we found that good performers spent less time on the return sweeps as they made larger saccades with fewer intermittent fixations to accomplish them. The significant contribution of return sweeps to the horizontal DEM time also explains why other studies have found that the horizontal subtest correlates stronger with reading performance than the vertical subtest and the DEM ratio^10^. Finally, we noticed poor, non-significant correlations of the return sweep parameters with age. Because return sweep times contribute to determining the DEM ratio, these findings partly explain why the DEM ratio showed a weaker correlation with age than the horizontal and vertical DEM times.

An advantage of our new stereoscopic eye-tracking methodology is that it simplifies the in vivo calibration to a one-point fixation task^23^. Even so, the success rates were limited, thereby limiting the statistical power of the eye movement analyses. For Pearson’s correlations and paired *t* tests with a large effect size (ρ = 0.5), and a power (1 − β) of 0.80 with a significance level (α) of 0.05, the sample sizes required is 26 and 27 participants, respectively^43,44^. This means that the 28 children for whom we had complete eye movements records in both subtests were enough to reliably detect and reject large effect sizes (with Type I and II errors of α = 0.05 and β = 0.2, respectively) but not medium to small effect sizes (ρ = 0.3 and ρ = 0.1). We did not adjust these results for multiple comparison because we did not want to miss any possible association between the DEM and oculomotor skills. Yet it is possible that some correlations between saccade parameters and vertical DEM performance are too weak to be picked-up in the present study.

Considering that eye-tracking equipment no longer needs to be expensive nor invasive, is the DEM still relevant for clinical diagnostics of oculomotor problems? An advantage of the DEM is its simplicity in administering the test. The comparison between horizontal and vertical DEM provides some indirect clues about oculomotor skills in healthy children, but the results do not warrant conclusions about possible pathology. To accurately diagnose oculomotor problems, abnormal DEM scores should be followed up by more specialized diagnostic measurements^45–49^. That said, horizontal and vertical DEM times are significantly associated with literacy and numeracy scores^13,14^, reading rate^15,16^, and speed of visual processing (this study and^10^). We therefore believe that the DEM offers a possibility to recognize anomalies in these domains at a relatively young age. For example, children with Cerebral Visual Impairments show delays in the processing of visual information^1,22^, that might also be reflected in reduced performance on the vertical and/or horizontal DEM.

Taken together, our results provide new insights in the age-dependent oculomotor behavior of children during the two subtests of the DEM. Saccade behavior is different between the horizontal and vertical DEM, but these differences do not warrant the conclusion that the DEM can assess saccade skills. Instead, the picture emerges that the oculomotor signature of DEM performance is visual fixation; a process linked to visual-verbal naming skills, visual processing speed, visual search strategies and other cognitive factors that guide fixation.

## Methods

### Participants

Ninety-one children (9.4 ± 2.0 years) were recruited. Inclusion criteria were age 5 to 12 years old, normal birth weight (> 2500 g), birth at term (> 36 weeks), no perinatal complications, no complaints of slow visual processing, crowded VA of 0.1 logMAR or better, and normal development. Testing occurred at the children’s primary schools. Children with glasses wore them during all tests. For details, see^21^.

Informed consent was obtained in writing from the parents of all participants. The study was approved by the local ethics committee Commissie Mensgebonden Onderzoek regio Arnhem-Nijmegen, The Netherlands (protocol NL48708.091.14), and conducted according to the principles of the Declaration of Helsinki.

### Developmental eye-movement test (DEM)

The DEM (Fig. 1a) was administered at ~ 65 cm. Children were asked to maintain that viewing distance without head restraint.

Children first practiced with a pre-test to familiarize them with the task, and to make sure that they could read numbers. The pre-test was a shortened version of each DEM subtest with randomized ordering of the numbers. Then, children had to name the numbers of DEM A from top to bottom, one column at a time. All numbers appeared on the computer screen as soon as the experimenter pressed the space bar and disappeared when the experimenter pressed the space bar again as soon as the child named the last number. These start and stop moments were recorded by the software. DEM A was followed by DEM B, which is similar to A but with the numbers in a different order.

The numbers of the DEM C array had to be named from left to right, starting at the top left. Horizontal time was taken as the total time to name the first to the last number of DEM C. For the list of applied numbers, see Supplementary Fig. 1 and^5^. High contrast (98.2% Michelson) numbers (2.1 cd/m^2^) of 4.9 mm in height (LogMAR 0.71, similar to the paper version of the DEM) were vertically spaced apart 14.6 mm against a white background (235.6 cd/m^2^). The two columns in DEM A and B were horizontally spaced apart 162.5 mm. For DEM C, the leftmost numbers and rightmost numbers were horizontally spaced apart 191.8 mm and spacing between numbers varied between 19.2 and 57.5 mm.

### Eye-tracking

Horizontal and vertical eye movements were measured with a remote stereoscopic eye tracking system^23^ consisting of two USB 3.0 cameras and two infrared light sources. The tracking software (http://github.com/Donders-Institute/Stereo-gaze-tracking) captured the pupil and corneal reflections (glints) of both eyes. The one-point calibration that is required for this method was part of another task^50^. The offline gaze reconstruction combined the asynchronous data from the two cameras into gaze position signals with an average refresh-rate of ~ 500 Hz. Since the system did not only record the point of gaze (POG) on the screen, but also the three-dimensional location of the eyes, we could accurately account for head translations. The spatial accuracy of the resulting eye movement measurements was ~ 0.7°. The spatial resolution was better than 0.2° and the sample-to-sample noise was less than 0.05°.

### Speed acuity test (SA)

The speed–acuity test^21^ was administered binocularly at 5 m. Each trial consisted of a high-contrast black Landolt-C presented at the center of the computer screen against a white background. Children had to indicate, as quickly and accurately as possible, on which side, right or left, the opening of the C was located by pressing the corresponding mouse button. The stimulus was presented until the child responded. Task difficulty was manipulated by presenting nine optotype sizes (ranging from 0.43 to 1.09 logMAR), each presented 10 times in pseudo random order.

Here, we only included the reaction times to the 0.68 LogMAR stimulus, which was similar to the size of the numbers of the DEM (0.71 LogMAR).

### Detection tasks

The children also performed a visual and an auditory detection task to measure the time children needed to respond to a supra-threshold stimulus^21^. In the visual detection task (20 trials), they had to press a mouse button as soon as they saw the visual stimulus (a large high-contrast black letter “O”). In the auditory detection task (20 trials), they had to press the mouse button as soon as they heard the sound (a 500 ms white noise burst of ~ 75 dBA).

### Equipment

The stimulus software was written in Matlab (version 2013b; MathWorks, Inc., Natick, MA, USA) using the Psychophysics Toolbox (version 3.0.12^51^). Stimulus timing and button presses were recorded and stored at 1-ms precision. The stimulus software was executed on a laptop (Dell M3800; Dell, Inc., Round Rock, TX, USA) equipped with an OpenGL graphics card (Nvidia Quadro K1100M; Santa Clara, CA, USA). The visual stimuli were presented on a 23-inch LCD screen (Dell, Inc. U2412M, 1920 × 1200 pixels, pixel pitch 0.27 mm). Visual stimulus properties were measured with a luminance meter (Minolta LS-100; Minolta Co. Ltd., Osaka, Japan). Ambient light conditions ranged from 100 to 350 lx as measured with a lux meter (Voltcraft MS-1500; Hirschau, Germany). Sound intensity was measured with a sound level meter (ISO-TECH SLM 1352P; ISO-Tech, Taipei, Taiwan) at the location of the subjects’ ears^21^.

The eye tracker consisted of two USB 3.0 cameras (Lumenera lt225 NIR, Lumenera Corp., Ottawa, Canada, pixel size 5.5 × 5.5 μm) connected to the stimulus laptop and two 850-nm infrared illuminators (Abus TV6700, ABUS KG, Wetter, Germany) mounted on an optic rail. The lenses with manual focus and diaphragm had a focal length of 16 mm (Navitar NMV-16M23, Navitar Inc, Rochester, NY, USA). Infrared-passing filters (UV/Vis-Cut R-72; Edmund Optics Inc., Barrington, NJ, USA) that passed wavelengths > 720 nm were added on the lenses to block light in the visible spectrum^23^.

### Procedure

Children first participated in the Freiburg visual acuity test to assess crowded VA (for details, see^21,52^), followed by SA, both administered digitally at 5 m distance. Subsequently, the computer screen was moved to 65 cm distance to measure eye movements during the DEM. For a small group of children, the DEM was performed on a different day. Test results for all children can be found in Supplementary table S2.

### Data analyses

The offline analysis was performed and images were created using Matlab (version 2018b).

#### DEM

Total vertical time was taken as DEM A time plus DEM B time. Time to complete DEM C was adjusted for omissions and additions^53^. Repeating a whole line counts as five addition errors. Skipping one-line counts as two omission errors. The number of errors was determined by adding the number of omissions and additions

$${\text{Adjusted}}\;{\text{ time}}\;{\text{ DEM}}\;{\text{ C }} = {\text{ Time }}\;{\text{test }}\;{\text{C }}*{ [8}0/\left( {{8}0 \, - {\text{ omissions }} + {\text{ additions}}} \right){]}$$

We used adjusted DEM C times for regression analyses without eye tracking, and raw DEM C times otherwise. The time for DEM A and B was not adjusted for errors because of the limited number of errors made during those tests.

#### Speed-Acuity

For SA and detection tasks, we computed mean reaction times after removing atypically long or short reaction times. Trials were excluded from the mean if the reaction time deviated more than three times the median absolute deviation (MAD) from the median after discarding reaction times < 0.1 s. Children whose mean reaction time deviated more than three standard deviations from the mean were excluded (excluded: 0/90 in SA, 2/90 in visual task, 1/90 in auditory task). Correlation coefficients were calculated in Matlab using Pearson’s correlation coefficient, and Pearson’s linear partial correlation with age as a confounding variable. Multiple comparisons were done using False Discovery Rate (FDR)^54^.

#### Eye-tracking

We used data from the eye with the best tracking signal. Data where signals for both eyes were lost were not included. The sampling rate of the gaze position signals was variable because the two cameras ran asynchronously^23^. Therefore, the data were resampled to a fixed sampling rate of 500 Hz using linear interpolation. Prior to this interpolation, a 13-point median filter was applied, followed by zero-phase filtering with a low-pass Butterworth filter (5th order, 40 Hz cut-off) after resampling.

Saccades were detected with custom software using velocity and acceleration threshold criteria for saccade onsets and offsets that were all set to 3 times the median absolute deviation (MAD) of the noise (typically around 20°/s and 3000°/s^2^ or less). Eye velocity was taken as the vector sum of horizontal and vertical eye velocities (track velocity). The acceleration signal was the time derivative of eye track velocity. Differentiation with respect to time was performed with a 9-point noise-robust differentiator. The software checked if eye positions measured in a 20 ms time window before saccade onset were significantly different from the eye positions measured in a 20 ms time window after saccade offset. All saccade markings were visually inspected and corrected if necessary.

Horizontal saccades were all saccades with a direction below ± 45° from the horizontal meridian. All other saccades were included as vertical saccades. We did not distinguish oblique saccades. Fixation duration was determined from the time between the offset of one saccade and the onset of the next saccade. Fixation time was defined as the sum of all fixation durations in a given time window. Saccade time was defined as the summed durations of all saccades in a given time window. Occasionally, the eye-tracking signal was briefly lost due to eye blinks, which lead to a relatively long, and incorrect, fixation duration between two saccades. Therefore, fixation durations > 3 * MAD from the median were removed from the dataset.

## Supplementary Information

Supplementary tables and figures

## Data Availability

The datasets generated during and/or analyzed during the current study are available from the corresponding author on reasonable request.
