# Supplementary material for: Visual fixations rather than saccades dominate the developmental eye movement test
Source: Sci Rep. 2021 Jan 13;11:1162. doi: 10.1038/s41598-020-80870-5 (PMC7806730; doi:10.1038/s41598-020-80870-5)
Supplement: Supplementary file 1 — Supplementary tables and figures [file 41598_2020_80870_MOESM1_ESM.pdf]

Visual Fixations rather than Saccades Dominate the Developmental Eye Movement test

Nouk Tanke,[1] Annemiek D. Barsingerhorn,[2] F. Nienke Boonstra,[1,3] Jeroen Goossens[1]*

1. Department of Cognitive Neuroscience, Donders Institute for Brain, Cognition and Behaviour, Radboud University Medical Centre, Nijmegen, the Netherlands.
2. Department of Biophysics, Donders Institute for Brain, Cognition and Behaviour, Radboud University, Nijmegen, the Netherlands.
3. Royal Dutch Visio, National Foundation for the Visually Impaired and Blind, Nijmegen, the Netherlands.

* corresponding author: J.Goossens@donders.ru.nl

Supplementary Figures and Tables

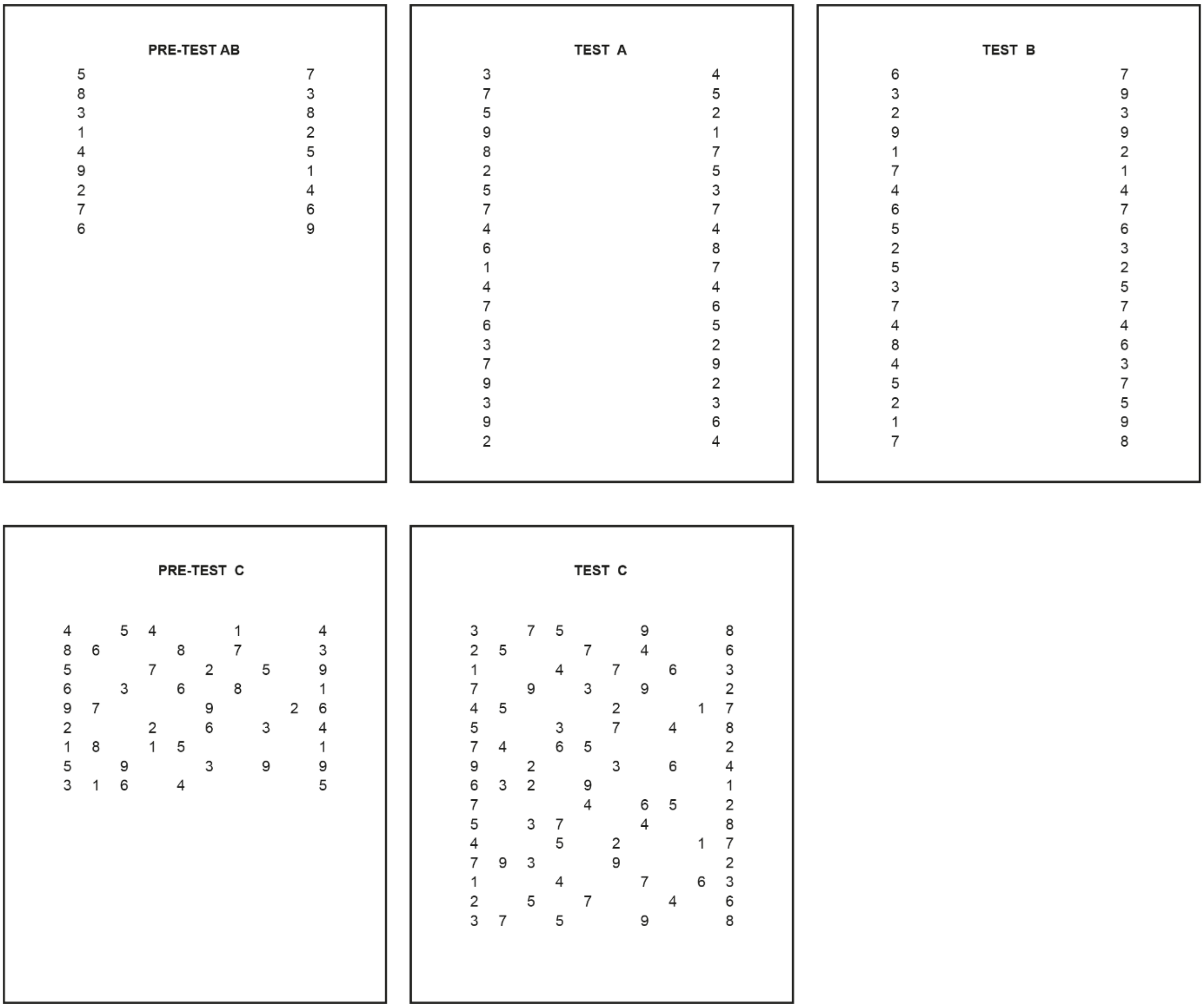

Supplementary figure 1. Overview of the numbers used in the DEM.
Complete overview of the number order of different sub-tests of the DEM task. Pre-test for AB, DEM A and DEM B are read vertically. Pre-test for C and DEM C are read horizontally. The number series in DEM A, B and C were always the same. Number ordering in the pretests was randomized between subjects. Produced in Adobe Illustrator CC 2017 (www.adobe.com).

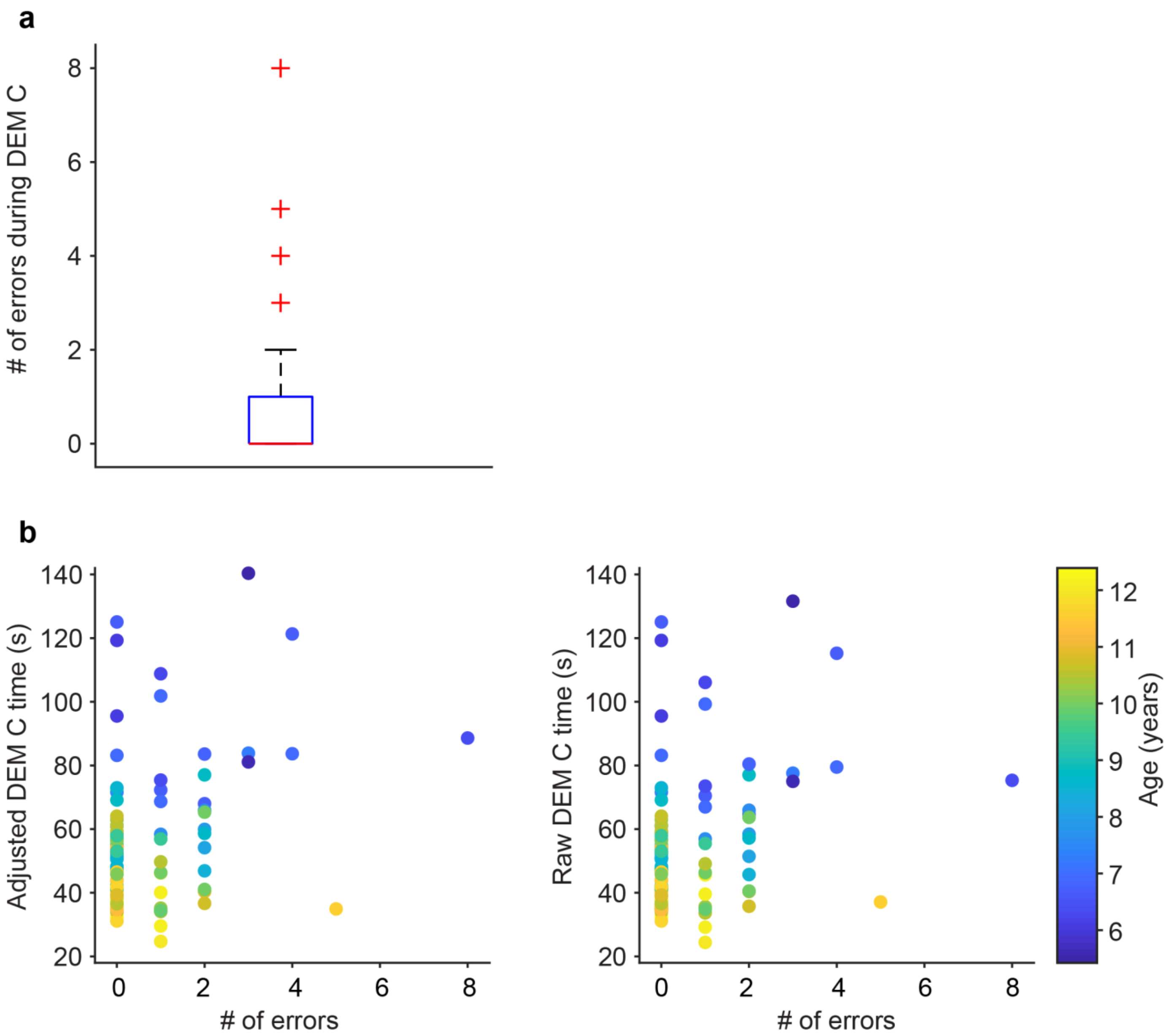

Supplementary figure 2. Errors made during horizontal DEM.
(**A**) Boxplot showing mean number of errors made during horizontal DEM. Red + indicate values above the 99th percentile. (**B**) Visual representation of number of errors per child and their score on the horizontal DEM, color-coded for age. Note, that younger children made more errors than older children did, and that children who made more errors spent a similar amount of time performing the horizontal DEM compared to age-matched children who made fewer errors. Note also the negligible difference between the plot for adjusted horizontal time (left) and raw horizontal time (right). Produced in MATLAB 2018b (www.mathworks.com).

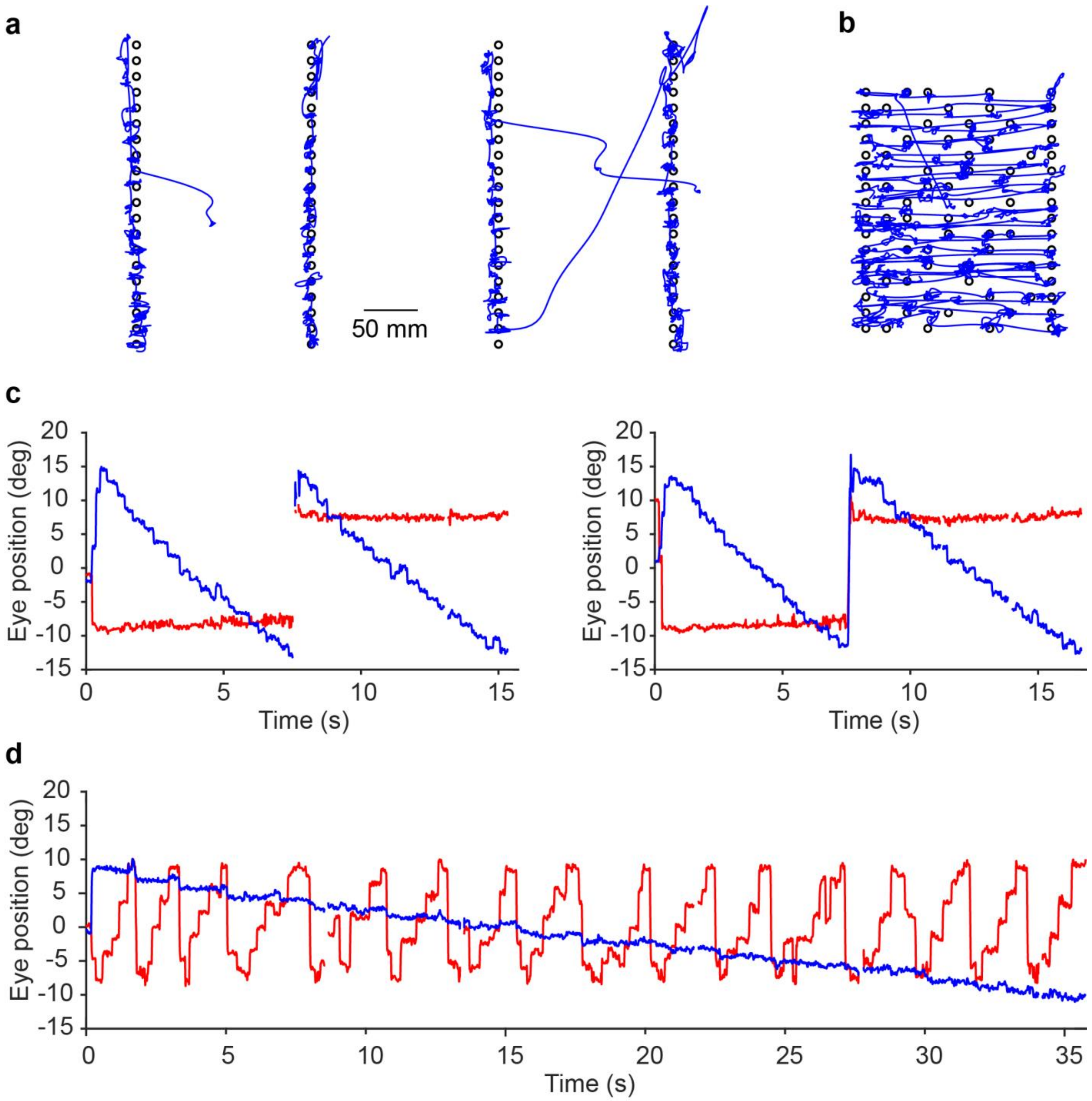

Supplementary figure 3. Eye movement traces during the vertical and horizontal DEM. Same subject as shown in **Fig. 2A**. Eye-tracking data for the right eye. (**A-B**) Point of gaze on the screen (blue lines) during the vertical DEM (**A**) and the horizontal DEM (**B**). Circles represent number locations. (**C-D**) Horizontal (red) and vertical (blue) eye positions over time during the vertical DEM (**C**) and the horizontal DEM (**D**). Produced in MATLAB 2018b (www.mathworks.com).

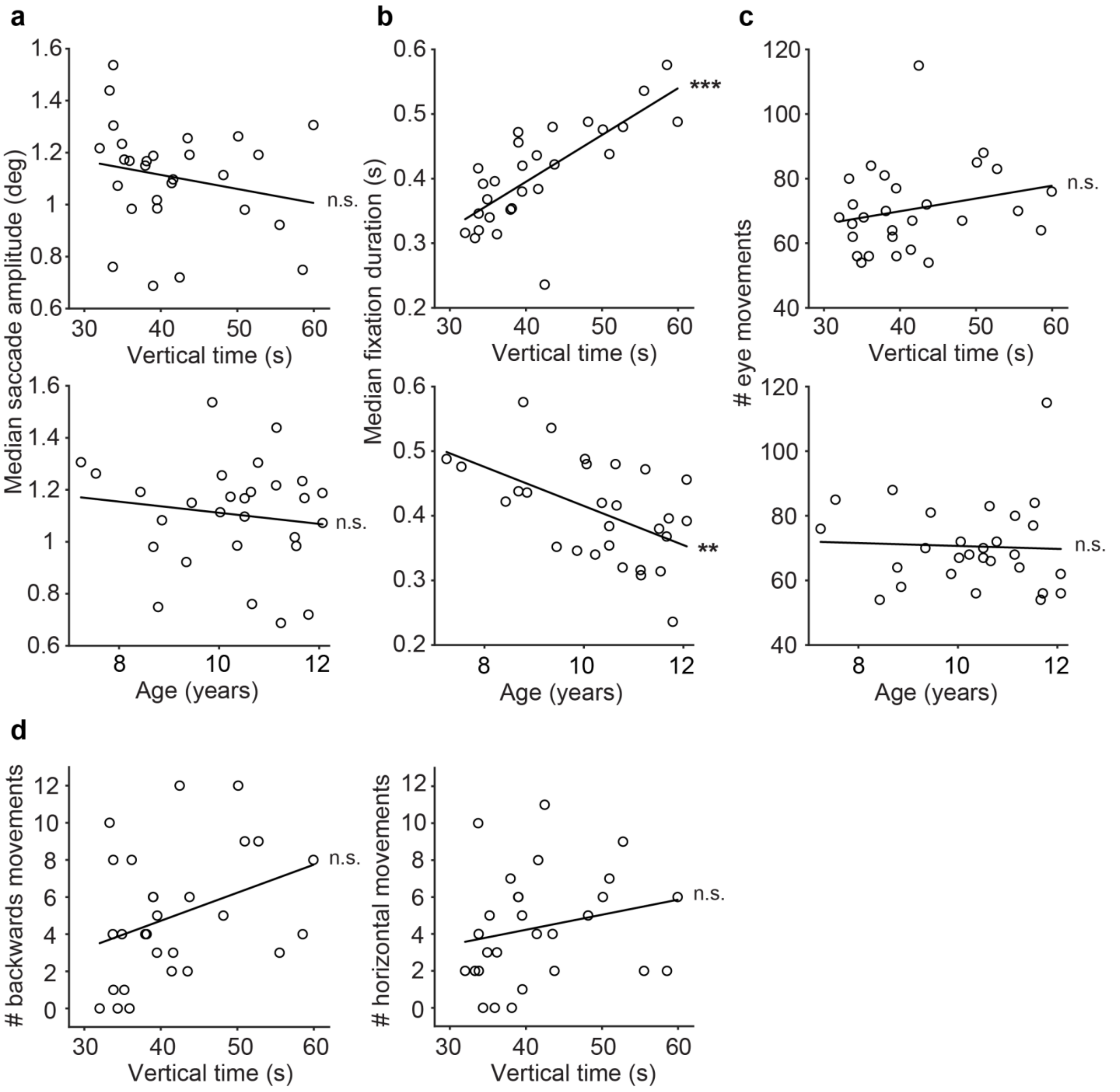

Supplementary figure 4. Saccade amplitude and eye movement counts during the vertical DEM
Same as figure 4, but for the vertical DEM. (**A**) Median saccade amplitude during number identification plotted against the vertical DEM completion time (up; r = -0.21, p = 0.28) and age (bottom; r = -0.13, p = 0.48). (**B**) Same as **A**, but for median fixation duration (DEM time, r = 0.75, p < 0.001; age, r = -0.52, p = 0.0049). (**C**) Same as **A**, but for number of eye movements (DEM time, r = 0.24, p = 0.22; age, r = -0.05, p = 0.82). (**D**) Number of backwards eye movements (left; r = 0.34, p = 0.069) and number of horizontal eye movements (right; r = 0.22, p = 0.26) during number identification in the vertical DEM. *** p < 0.001, ** p < 0.01, n.s. = not significant**.** Produced in MATLAB 2018b (www.mathworks.com).

Supplementary table 1. Average DEM scores per age group in participants with eye tracking during the horizontal DEM. Horizontal DEM times are adjusted for the number of errors. Naming is vertical DEM time (in sec) as a percentage of horizontal DEM time (in sec).

Age	Vertical DEM mean (SD)	Horizontal DEM mean (SD)	DEM ratio mean (SD)	Errors mean (SD)	Naming mean (SD)
6 (n = 0)					
7 (n = 4)	54.24 (6.94)	69.34 (11.10)	1.28 (0.08)	1.50 (1.29)	79 % (4.89)
8 (n = 5)	48.65 (5.42)	54.51 (12.58)	1.11 (0.13)	1.20 (1.10)	91 % (10.43)
9 (n = 4)	43.78 (9.75)	49.47 (14.15)	1.13 (0.17)	1.00 (0.82)	90 % (11.82)
10 (n = 9)	40.40 (6.82)	44.41 (10.39)	1.09 (0.10)	0.44 (0.73)	92 % (9.37)
11 (n = 12)	37.61 (5.53)	39.62 (6.48)	1.06 (0.13)	0.42 (1.44)	96 % (10.92)
12 (n = 2)	35.81 (4.53)	41.73 (6.34)	1.16 (0.03)	0.50 (0.71)	86 % (2.21)

Supplementary table 2. Scores on each test per child. Grey background indicates successful eye tracking during different subtasks of the DEM. DEM C times are adjusted for errors.

Subject	Age (years)	VA (LogMAR)	SA (s)	DEM A (s)	DEM B (s)	DEM C (s)
1	12.39	-0.30	0.49	16.95	19.68	32.42
2	11.46	-0.30	0.54	21.35	22.59	43.77
3	11.79	-0.30	0.53	22.15	20.31	40.41
4	11.98	-0.24	0.62	22.08	22.61	47.79
5	11.14	-0.30	0.54	15.24	16	34.81
6	12.19	-0.21	0.70	18.2	18.45	40.18
7	11.15	-0.30	0.74	15.18	16.65	41.05
8	10.73	-0.30	0.46	18.79	16.36	37.56
9	10.23	-0.27	0.61	18	17.22	35.17
10	11.09	-0.26	0.59	21.71	23.08	41.36
11	12.07	-0.07	0.46	19.29	19.72	46.21
12	10.70	-0.30	0.56	20.67	21.51	41.59
13	10.47	-0.30	0.50	16.35	19.02	34.11
14	10.76	-0.15	0.53	14.18	17.17	33.93
15	9.91	-0.30	0.66	22.9	21.14	52.06
16	9.45	-0.30	0.71	17.44	20.53	41.26
17	9.76	-0.16	0.69	23.19	22.91	53.86
18	9.91	-0.20	0.59	17.7	18.01	34.9
19	9.86	-0.30	0.55	16.89	16.99	34.41
20	9.72	0.03	0.72	17.2	18.91	35.19
21	9.93	-0.30	0.61	21.72	20.9	47.07
22	9.36	-0.30	0.68	30.4	35.63	55.03
23	9.23	-0.19	0.61	25.58	30.14	56.78
24	8.61	-0.23	0.55	20.64	20.61	47.45
25	7.65	-0.22	0.75	21.48	24.04	51.09
26	7.88	-0.24	0.75	25.06	30.11	59.91
27	7.45	-0.20	0.79	29.4	30.5	71.59
28	7.72	-0.24	0.80	28.41	28.53	61
29	8.42	-0.24	0.85	22.06	23.25	50.83
30	8.78	-0.14	0.68	29.27	27.61	74.26
31	9.37	-0.30	0.72	24.21	29.71	58.85
32	8.56	-0.09	0.64	18.99	18.62	48.26
33	8.25	-0.29	0.74	21.56	22.82	52.58
34	8.49	-0.30	0.64	23.9	23.38	50.54
35	8.86	-0.30	0.57	20.71	23.65	42.2
36	8.43	-0.20	0.67	21.22	22.53	46.89
37	6.78	-0.29	0.80	38.54	35.5	83.58
38	6.95	-0.24	1.10	28.74	35.37	101.84
39	6.70	-0.30	0.90	46.3	56.83	125.07
40	6.71	-0.25	0.78	34.08	32.07	67.98
41	6.26	-0.08	1.20	34.07	40.05	108.8
42	7.54	-0.21	0.68	25.82	24.29	63.55
43	6.65	-0.18	0.77	36.08	38.62	116.7
44	6.63	-0.21	0.83	32.19	38.32	72.29

45	7.24	-0.22	0.79	30.36	29.97	83.86
46	6.88	-0.14	0.98	33.13	33.67	83.17
47	6.86	-0.20	0.84	32.46	30.56	80.5
48	6.37	-0.20	0.85	32.18	37.96	75.4
49	5.62	-0.30	0.99	33.45	31.92	81.1
50	5.42	-0.22	1.01	69.02	67.87	140.38
51	5.98	-0.27	0.89	42.34	47.27	119.28
52	6.00	-0.24	1.08	38.83	39.89	95.54
53	6.40	-0.29	0.87	38.91	48.29	88.61
54	6.94	-0.24	0.89	33.62	33.46	68.69
55	9.91	-0.20	0.63	20.56	22.56	46.33
56	11.10	-0.16	0.68	23.97	24.93	54.32
57	12.15	-0.30	0.58	19.35	21.91	40.08
58	12.07	-0.30	0.58	15.42	17.18	37.24
59	11.59	-0.30	0.56	17.57	17.9	35.99
60	10.66	-0.22	0.61	15.72	16.86	36.67
61	11.96	-0.29	0.51	11.28	12.45	24.71
62	12.15	0.01	0.82	13.22	14.02	29.55
63	11.73	-0.21	0.60	17.99	18.08	31.13
64	10.91	-0.21	0.63	25.67	20.44	38.91
65	11.54	-0.22	0.56	17.13	19.06	34.92
66	10.80	-0.30	0.47	15.59	16.98	38.95
67	10.36	-0.30	0.68	19.74	19.79	35.98
68	11.66	-0.23	0.47	17.64	17.28	35.54
69	11.51	-0.29	0.48	18.23	21.26	44.05
70	11.24	-0.11	0.63	15.97	19.49	34.72
71	11.71	0.00	no data	15.43	17.95	41.87
72	11.56	-0.26	0.57	20.19	25.77	46.53
73	10.61	-0.18	0.65	23.67	25.2	59.34
74	10.23	-0.23	0.75	23.76	24.99	62.94
75	10.50	-0.15	0.74	21.24	22.29	61.18
76	10.05	-0.29	0.57	21.84	21.66	45.83
77	10.51	-0.28	0.62	17.37	19.07	36.58
78	10.51	-0.30	0.56	18.9	22.71	49.72
79	10.02	-0.25	0.68	22.7	25.47	56.35
80	10.64	-0.19	0.64	26.28	26.48	64.07
81	10.78	-0.28	0.53	16.24	17.56	39.3
82	9.90	-0.30	0.66	17.26	19.03	41.05
83	8.73	-0.30	0.85	29.16	30.51	69.15
84	8.49	-0.22	0.82	38.04	36.23	72.97
85	8.12	-0.13	0.97	23.23	25.77	54.16
86	7.58	-0.19	0.85	24.62	21.98	58.37
87	9.35	-0.24	0.76	23.6	31.92	56.9
88	9.90	-0.29	0.66	24.2	23.54	65.32
89	8.68	-0.30	0.80	22.47	28.5	58.66
90	9.42	-0.30	0.68	22.98	28.3	53.05
91	9.44	-0.30	0.61	26.74	23.38	57.92
